# 

**Published:** 1923-03

**Authors:** 


**THE DENTAL REGISTER** is published every month by the Samuel A. Crocker Company, 18 W. 7th Street, Cincinnati, O. It is mailed to subscribers during the last week in each month, being a month-end publication. Have copy for current month here by the 15th of each month. Copy received by the 15th of the month goes into that month's number of the Dental Register. Publication and business office, 18 W. 7th Street, Cincinnati, Ohio. Editorial office, 18 W. 7th Street, Cincinnati, Ohio. Address all correspondence to Editor Dental Register, Box 838, Cincinnati, Ohio, c'o The Samuel A. Crocker Company.

Notices of meetings, announcements of interest to the dental profession, and particulars of new ideas and new inventions will be given free publication in reading section. Advertising section is open to all ethical advertisements.

We are not responsible for opinions or statements made by writers whose articles this magazine may publish—nor does the publishing of any article infer in any way that we indorse the views or opinions expressed by any writer whose article we may publish.

### Sal Hepatica

Materially Aids

Local Treatment

IN

PYORRHEA

Samples on Request

Bristol-Myers Co.
NEW YORK

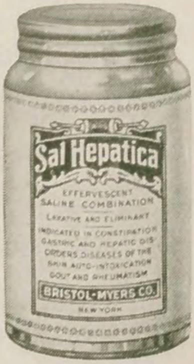

### DIRECTORY

FOR

### Members of the Dental Profession whose practices are limited to a specialty.

The object of this directory is to furnish ready reference to specialists who take referred patients. If you wish your name inserted in this list please correspond with the Dental Register, Box 838, Cincinnati, Ohio.

Your name, specialty, office hours and phone number will be published.

This entire page is reserved for Professional Directory.

### Cincinnati

Dental X-Ray Diagnosis
CHARLES K. FIELD
61 Groton Bldg., 7th and Race Sts.
Hours, 10 to 4
Canal 2127

Periodontoclasia (Pyorrhea and Oral Prophylaxis)
EDGAR H. EBERLE
605-06 Union Central Bldg., 4th and Vine Sts.
Hours, 9 to 5—Consultation hours by appointment
Main 882

Periodontoclasia (Pyorrhea and Oral Prophylaxis)
MARIE L. BECKERT
25 Groton Bldg., 7th and Race Sts.
Hours, 9 to 5—Consultation hours by appointment
Canal 842

Exodontia (Extraction and Oral Surgery)
ROBERT M. SCHELL
20 W. 9th St., between Vine and Race Sts.
Hours, 1 to 5—Consultation by appointment
Canal 118

### ANNOUNCEMENTS

THE AMERICAN ACADEMY OF PERIODONTOLOGY

The Tenth Annual Meeting of the American Academy of Periodontology will be held at the Hotel Statler, Cleveland, Ohio, September 7 and 8, 1923; just preceding the convention of the American Dental Association, in Cleveland, from September 11th to 14th inclusive.

The Sections of Orthodontia and Peridontia will conduct its meetings in the Ball Room of the Hotel Statler the week of September 11th, making it convenient for Academy members to attend these meetings.

The 1923 meetings of both the Academy and the American Dental Association promise to be the largest in the history of the organizations and it will be necessary for you to make reservations immediately with the Hotel Statler and state that you desire your reservations to continue through the week of the American Dental Association meeting. These reservations will be acknowledged by the Hotel, on special blanks provided for that purpose. This will assure you accommodations and prevent confusion on your arrival.

The Academy meeting will be called to order Friday, September 7th, at 10:00 A. M. in the Ball Room of the Hotel Statler. The Program Committee has already secured some of the best talent of the Medical and Dental professions for this meeting.

This being the Tenth meeting and held in Cleveland, the City where the Academy was organized, the Officers and Members of the Council are desirous of having every member attend this 1923 meeting.

You will receive further information as soon as the Program Committee and the Local Arrangements Committee has completed all the details for the meeting.

Write for accommodations to the Hotel Statler or one of the following hotels: Hotel Cleveland, Hotel Winton, Hollenden Hotel.

---

NOTICE THE DATE

The fifty-fourth annual meeting of the Kentucky State Dental Association will be held in Louisville, Kentucky, April 16-19, 1923.

Slogan—"Be a Booster." Headquarters—Seelbach Hotel.

*Officers*—E. C. Hume, President, 814 Francis Bldg., Louisville; N. B. Smith, Vice-President, Frankfort; R. L. Spratt, Treasurer, Mt. Sterling; W. M. Randall, Secretary, 1035 S. Second St., Louisville.

*Executive Committee*—R. L. Sprau, 968 Baxter Ave., Louisville; W. E. Goepper, Louisville; Chas. Pearcy, Louisville.

*Master of Exhibits and Editor of Program*—Geo. H. Means, Cherokee Apartments, Louisville.

*Board of Trustees*—E. C. Hume, President ex-officio; W. M. Randall, Secretary ex-officio; R. L. Spratt, Treasurer ex-officio; W. F. Walz, Bd. of Ex. ex-officio; W. A. Bridwell, '25; J. E. Faulkner, '25; C. H. Tandy, '25; E. P. Marcilliat, '24; P. H. Williams, '24; Guy Clark, '24; Walter Matthews, '23; W. G. Best, '23.

*Dento-Legal Committee*—I. H. Harrington, Chairman, Louisville, Ky.; W. M. Randall, Louisville, Ky.; R. L. Spratt, Mt. Sterling, Ky.

*Clinic Committee*—J. H. Fullenwider, Chairman, Louisville, Ky.; A. S. Ingman, Louisville, Ky.; Joe Seldon, Louisville, Ky.; Chas. Mathis, Louisville, Ky.; J. B. Robarts, Louisville, Ky.; A. T. Wooten, Central City, Ky.; C. P. Mayhall, Harlan, Ky.

*Ethical Committee*—Oscar Flener, Chairman, Hopkinsville, Ky.; H. G. Hazelrig, Paintsville, Ky.; M. B. Guthrie, Lexington, Ky.

*Oral Hygiene Committee*—O. D. Wilson, Chairman, Owensboro, Ky.; J. F. Nevitt, Lexington, Ky.; G. H. Heyman, Louisville, Ky.

*Dental Research and Invention Committee*—O. B. Powell, Paducah, Ky.; Frank Hower, Louisville, Ky.; H. J. Patrick, Paint Lick, Ky.

*Auditing Committee*—R. P. Keene, Chairman, Owensboro, Ky.; C. J. Zimmer, Lexington, Ky.; H. L. Duncan, Dixon, Ky.

*Transportation Committee*—Joe Jones, Chairman, Dawson Springs, Ky.; O. B. Heaverin, Owensboro, Ky.; X. E. Sanders, Bowling Green, Ky.

*Press Committee*—Harry Kettig, Chairman, Louisville, Ky.; J. H. Gunkle, Newport, Ky.; W. T. Hays, Providence, Ky.; J. H. Feamster, Frankfort, Ky.

*Necrology Committee*—Power Wolfe, Chairman, Princeton, Ky.; C. A. Sanders, Perryville, Ky.; W. G. Best, Berea, Ky.

*Membership Committee*—E. P. Marcilliat, Chairman, Starks Bldg., Louisville, Ky.; All secretaries of component societies.

*Publication Committee*—W. M. Randall, Chairman ex-officio, Louisville, Ky.; W. W. Burgin, Campbellsville, Ky.; T. W. Lander, Eddyville, Ky.

*Entertainment Committee*—A. P. Williams, Chairman, Louisville, Ky.; H. Nichols, Louisville, Ky.; R. P. Thomas, Louisville, Ky.; A. T. Mock, Louisville, Ky.

*Science and Literature Committee*—Hugh McElrath, Chairman, Murray, Ky.; C. R. Ellis, Shelbyville, Ky.; S. G. Walton, Newport, Ky.

*Board of Censors*—E. B. Hardin, Chairman, Madisonville, Ky.; J. O. McCawley, Sturgis, Ky.; T. D. Kelly, Jr., Lexington, Ky.

*Legislative Committee*—E. D. Rose, Chairman, Bowling Green, Ky.; Others to be announced later.

The fifty-fourth annual meeting of the Kentucky State Dental Association which takes place April 16-19, 1923, is going to be a banner convention for this organization. All the committees, which comprise many of the prominent dentists of the state, are working hard to make the meeting interesting and attractive to the profession so that all who attend the clinics and meeting this year will be repaid for their visit to Louisville and can take home new enthusiasm and valuable, up-to-date information of the progress of their great calling. The exhibits will be particularly interesting and there will be exhibited the latest in specialties and fixtures besides the standard supplies with which the dentists are so familiar. The get-together spirit is going to be a feature of the meeting and it is earnestly desired by the executive heads of the various committees that all Kentucky dentists set aside April 16th to 19th for a trip to Louisville. Reservations at the Hotel should be made now.

---

The annual meeting of the West Virginia State Dental Society will be held at Wheeling, W. Va., May 21-23, 1923, in the Market Auditorium Building.

A large attendance is expected and it is hoped to make this the best meeting ever held in the State. There is an excellent room for the exhibitors, which will be held both day and night, with every convenience. 110 V. A.C. for those who will need electricity.

The sessions of the society will be held in a separate room, so there will not be any conflict between meetings and exhibitors.

One evening will be given entirely to the exhibitors, no meeting at that time.

---

FIFTY-FOURTH ANNUAL MEETING KENTUCKY STATE DENTAL SOCIETY, LOUISVILLE, KY., APRIL 16-19, 1923

With every preliminary arrangement completed and with a full program made up, the officers and executive committee of the Kentucky State Dental Society are eagerly awaiting the opening of the fifty-fourth annual meeting of the association, confident that the dentists of Kentucky are going to enjoy, this year, the most interesting and instructive as well as the pleasantest convention in the history of the organization.

The main meetings of the Dental Association, the clinics and the group sessions will all be held in the Seelbach Hotel, which will be headquarters during the four days of the convention. Numerous entertainment features have been planned to furnish recreation for the visitors after the working hours of the convention each day, including dinners, receptions, sight-seeing tours and private events.

Despite the fact that members of the State Dental Association are Kentuckians and that the large majority are familiar with the city of Louisville, visitors this year will find a hitherto unfamiliar atmosphere about the metropolis of the state—the hustling air of progress usually associated only with the boom towns of the West.

Visitors in Louisville will find evidences of extreme activity on every side, the greatest proof of Louisville's rapid growth being the enormous amount of building that is going on. Building operations for the past January, for instance, increased 600 per cent., or by nearly $3,000,000, over January, 1922, most of the construction being of homes. Among the larger buildings now being built are the new Brown Hotel, $3,500,000, at Fourth and Broadway, to be finished next fall; the Elks' Home, $1,500,000, to be completed next January; a new baseball park, new interurban station and literally dozens of new factory and commercial buildings.

Having been carried through the recent industrial and commercial depression with scarcely a touch of hard times, because of her great intrinsic prosperity and her wide diversification of industries, Louisville's business is now growing with intense rapidity. Today the city can boast of more than fifteen plants, representing fifteen separate and distinct lines of manufactures, which are the largest of their kind in the world, while other factories are constantly being attracted to Louisville by the excellence of her location and the convenient supply of raw materials.

But not everything about Louisville is bustle, visitors to the Kentucky State Dental Association's meeting will find. There will be a wide-armed, whole-hearted welcome from the local membership of the association, as well as from the people of the city. Louisville, too, can show visitors the finest park system in North America, containing two parks which experts have pronounced the most beautiful in the world.

April, the time of the dental convention, is, as every Kentuckian knows, the most wonderful time of the year in the Blue Grass State—the time when every tree is in full new leaf, when the fruit trees and the flowering shrubs are in full bloom. State dentists will have a chance to see Louisville's parks and her lovely shaded drives in the height of their springtime glory.

---

### REHWINKEL DENTAL SOCIETY

The twelfth annual mid-winter meeting of the Rehwinkel Dental Society, was held Saturday, March 17, 1923, in the Masonic Temple Building, Chillicothe, O. Exhibits were open throughout the day.

1:00 P. M., Registrations, Dr. Johns. Galbraith, Registrar,. Business and report of secretary and treasurer.

*Clinics*—1. Resume of Dr. Cummer's Classification of Partial Denture Construction, Dr. J. V. Gentilly, Cleveland; 2. Office Helps, Dr. W. A. Loope, Cleveland; 3. Another Method of Testing the Vitality of a Pulp, Dr. E. L. Pettibone, Cleveland; 4. Fine Points in X-Ray Reading, Dr. George Kalb, Columbus; 5. Prosthetic Clinic, Dr. C. T. Boyd, Columbus; 6. Porcelain Jacket Crowns, Dr. C. R. Marshall, Columbus; 7. Repairing Bridges with Pin Facings, Dr. Oscar Miesse, Columbus; 8. X-Ray Interpretation, Dr. C. K. Fields, Cincinnati; 9. Bridge Work, Dr. F. H. Starr, Columbus; 10. Hobbies, "Non Dental," Dr. J. M. Henderson, Columbus; 11. Tooth Forms, Dr. E. W. Strosnider, Columbus; 12. Partial Denture, Dr. H. V. Cottrell, Columbus; 13. Government Dental Work, Dr. Elmer E. Stiner, Columbus; 14. Dental Research Laboratory, Dr. L. E. Custer, Dayton; 15. Some Alloy Fillings, etc., Dr. J. O. Hawkins, Wellston.

*Papers*—"Diagnosis of Disturbances of Oral Cavity," Dr. W. H. O. McGeehe, Cleveland; "Novocain Dermatitis," Dr. D. P. Snyder, Columbus.

*Exhibitors*—Samuel A. Crocker Co., Phillips Chemical Co., Dayton Dental Supply Co., Luthol Research Co., Medical Protective Co., Colgate & Co., The J. A. Sprague Co., Leibel Florsheim.

We were honored by having our State Officers and Committeemen on our program this year. They announced the State Program, Clinical Contest and the progress of the State Oral Hygiene work. The Ad-Interim Committee of the Ohio State Dental Society was also present.

---

### CINCINNATI DENTAL SOCIETY

The regular meeting of the Cincinnati Dental Society was held Friday, March 16th, 8:00 P. M., at the Literary Club Rooms. The following program was given:

*Essayist*—Geo. V. I. Brown, M. D. Subject: "Plastic and Oral Surgical Principles as Exemplified in the Treatment of Harelip, Cleft Palate and other Facial and Oral Restorations."

*Discussion*—C. A. Langsdale, M. D.; Chas. T. Souther, M. D.; M. R. Reid, M. D.

The following applications for membership were received at the last meeting: H. E. Burnett, Spring Grove & Hoffner; T. H. Arnold, 3901 Harrison Ave.

R. C. HARKRADER, Corresponding Secretary.

